# Comparison of Regenerative Tissue Quality following Matrix-Associated Cell Implantation Using Amplified Chondrocytes Compared to Synovium-Derived Stem Cells in a Rabbit Model for Cartilage Lesions

**DOI:** 10.1155/2018/4142031

**Published:** 2018-04-19

**Authors:** Hagen Schmal, Justyna M. Kowal, Moustapha Kassem, Michael Seidenstuecker, Anke Bernstein, Katharina Böttiger, Tanshiyue Xiong, Norbert P. Südkamp, Eva J. Kubosch

**Affiliations:** ^1^Department of Orthopedics and Trauma Surgery, Medical Center-Albert-Ludwigs-University of Freiburg, Faculty of Medicine, Albert-Ludwigs-University of Freiburg, Freiburg, Germany; ^2^Department of Orthopaedics and Traumatology, Odense University Hospital and Department of Clinical Research, University of Southern Denmark, Odense, Denmark; ^3^Molecular Endocrinology & Stem Cell Research Unit (KMEB), Department of Endocrinology and Metabolism, Odense University Hospital and University of Southern Denmark, Odense, Denmark

## Abstract

Known problems of the autologous chondrocyte implantation motivate the search for cellular alternatives. The aim of the study was to test the potential of synovium-derived stem cells (SMSC) to regenerate cartilage using a matrix-associated implantation. In an osteochondral defect model of the medial femoral condyle in a rabbit, a collagen membrane was seeded with either culture-expanded allogenic chondrocytes or SMSC and then transplanted into the lesion. A tailored piece synovium served as a control. Rabbit SMSC formed typical cartilage in vitro. Macroscopic evaluation of defect healing and the thickness of the regenerated tissue did not reveal a significant difference between the intervention groups. However, instantaneous and shear modulus, reflecting the biomechanical strength of the repair tissue, was superior in the implantation group using allogenic chondrocytes (*p* < 0.05). This correlated with a more chondrogenic structure and higher proteoglycan expression, resulting in a lower OARSI score (*p* < 0.05). The repair tissue of all groups expressed comparable amounts of the collagen types I, II, and X. Cartilage regeneration following matrix-associated implantation using allogenic undifferentiated synovium-derived stem cells in a defect model in rabbits showed similar macroscopic results and collagen composition compared to amplified chondrocytes; however, biomechanical characteristics and histological scoring were inferior.

## 1. Introduction

Articular cartilage defects often result in pain, loss of function, and finally osteoarthritis (OA), which cause a significant impact to the public health system in every developed country, where OA currently affects one in eight individuals [1]. Autologous chondrocyte implantation is a cellular therapy, which has successfully been employed to treat large, isolated, full thickness cartilage defects [2]. Several disadvantages such as the need for two surgical procedures and a significant donor site morbidity underline the need for modifications of the procedure. Furthermore, typical complications such as formation of hypertrophic regenerative cartilage, disturbed bonding of repair cartilage, insufficient biomechanical resistance of the newly formed cartilage, and delamination [3] drive the search for alternative techniques. Mesenchymal (stromal) stem cells, particularly synovium-derived mesenchymal stem cells (SMSC), represent a promising alternative cell source. This was concluded from their marker profile expressed on the cell surface [4, 5], indicating a chondrogenic phenotype, and their natural ability to form cartilage especially in the vicinity of chondrocytes [6]. Furthermore, the formation of hypertrophic differentiation was significantly less pronounced compared to that formed by bone marrow mesenchymal stem cells [7, 8].

SMSC is available in a high quantity and their procurement does not lead to significant donor site morbidity. The cellular characteristics of SMSC suggest their suitability for cartilage regeneration protocols based on their chondrogenic phenotype [5] including its maintenance after several cell culture passages and their excellent ability to form extracellular matrix [9]; however, how SMSC should be applied to cartilage defects to reach best repair quality needs to be determined.

Following this clinical paradigm, in the present study, we hypothesized that undifferentiated SCMC can repair cartilage lesions in a rabbit model of medial condyle full-thickness lesions just as efficient as allogenic culture-expanded chondrocytes. By using an allogenic transplantation approach, the study design is relevant to clinical application and mimics an off-the-shelf protocol [10]. The primary outcome criterion was biomechanical stability, the secondary outcome criterion the histological evaluation of repair quality. Explorative outcomes were the immunohistological evaluation of the expression of the collagen types I, II, and X, markers for chondrocyte differentiation and hypertrophy.

## 2. Methods

### 2.1. Cell Preparation

We followed the methods of Kubosch et al. [6].

Two animals were sacrificed 3 months before the experiments and the knees dissected totally removing the cartilage from the tibia and femur. At the same time, the knee synovia was prepared. The cartilage was cut into small pieces, washed, and transferred into DMEM F-12 10% (Lonza BioWhittaker, Basel, Switzerland), fetal calf serum (FCS), 1% penicillin/streptomycin (P/S) (Invitrogen, Karlsruhe, Germany), 0.5% gentamycin and 3% collagenase CLS type II (Biochrom, Berlin, Germany). Minced cartilaginous tissue was then enzymatically digested during the next 16 hours on a shaking incubator at 37°C with 200 rpm. Subsequently, the released chondrocytes were centrifuged, washed, and seeded in expansion medium DMEM F-12 supplemented with 10% FCS, 1% P/S, and 0.5% gentamycin. Expansion of chondrocytes was performed by seeding them on coated T-flasks with a density of 2500–5000 cells/cm^2^. The cells were frozen after reaching confluence. Thawed cells were grown and used when reaching a log phase of growth (passage 2). Similarly, the synovial tissue was cut into small pieces, washed, and transferred into DMEM F-12 medium with 10% FCS (Biochrom, Berlin, Germany), 1% penicillin/streptomycin (P/S) (Invitrogen, Karlsruhe, Germany), 0.5% gentamycin (Biochrom, Berlin, Germany), and 3% collagenase P (Roche, Mannheim, Germany). The suspension was digested during the next four hours on a shaking incubator (200 rpm) at 37°C. Subsequently, the released cells were centrifuged, washed, and seeded in expansion medium DMEM F-12 (10% FCS, 1% P/S, and 0.5% gentamycin). SMSC were seeded on coated T-flasks with a density of 2500–5000 cells/cm^2^ for expansion. The cells were frozen after reaching confluence. Thawed cells were grown and used when reaching a log phase of growth (passage 2). Both SMSC and chondrocytes were amplified, growth synchronized, and used for the animal experiments at passage 2.

### 2.2. Characterization of Rabbit Synovium-Derived Mesenchymal Stem Cells (SMSC)

#### 2.2.1. Chondrogenic Differentiation

Rabbit SMSC were distributed in 15 ml polypropylene tubes using 250,000 cells per 500 *μ*l medium (DMEM high glucose) supplemented with 10% FBS, 1% penicillin/streptomycin, 10% ITS (mixture of insulin, human transferrin, and sodium selenite from Corning, NY, USA), 1% sodium pyruvate (Thermo Fisher Scientific, MA, USA), 100 nM dexamethasone (Sigma-Aldrich, Brøndby, Denmark), 10 ng/ml TGF*β*3 (PeproTech, NJ, USA), and 50 μg/ml vitamin C (Sigma-Aldrich). In order to facilitate aggregate formation, the cells were gently centrifuged using 500*g* for 5 min. Chondrogenic media were changed every 2-3 days for 21 days, and cells were cultured in 37°C and 5% CO_2_.

#### 2.2.2. Osteogenic Differentiation

Rabbit SMSC were plated in density of 20,000 cells/cm^2^ in standard DMEM (low glucose) media and cultured until they reached 80–90% confluency. After 24 h, rabbit SMSC were exposed to osteogenic media described by Lee et al. [11], which contained DMEM (5.5 mM glucose), 10% FBS, 1% penicillin/streptomycin, 10 mM *β*-glycerophosphate (Calbiochem-Merck, Darmstadt, Germany), 100 nM dexamethasone, and 50 *μ*g/ml vitamin C (Sigma-Aldrich). Osteogenic media were changed every 2-3 days for 21 days, and cells were cultured in 37°C and 5% CO_2_.

*(1) Alkaline Phosphatase (ALP) Activity and Cell Viability*. ALP activity was measured at day 21 of osteogenic differentiation and normalized to cell viability. In order to asses cell viability, the cells were incubated with 20 *μ*l of CellTiter-Blue reagent (Promega, Mannheim, Germany) and 100 *μ*l media in 37°C for 1 h. After 1 h, the fluorescent intensity was measured (560_ex_/590_em_) in FluoStar Omega microplate reader (BMG Labtech, Birkerød, Denmark). Subsequently, the cells were washed with Tris-buffered saline and fixed with a mixture of 3.7% formaldehyde and 90% ethanol for 30 seconds at room temperature. After that, the cells were incubated with ALP substrate, 1 mg/ml p-nitrophenyl phosphate in 50 mM NaHCO_3_ (pH = 9.6) and 1 mM MgCl_2_ at 37°C for 20 min. The reaction was stopped by adding 3 M NaOH. Absorbance was measured at 405 nm using the FluoStar Omega microplate reader.

*(2) Alizarin Red Staining*. In order to assess matrix mineralization, after 21 days of osteogenic differentiation, rabbit SMSC were stained with Alizarin Red. Briefly, the cells were washed with PBS and fixed with 70% iced-cold ethanol for 1 h at −20°C. Subsequently, the cells were washed with H_2_O and stained with Alizarin Red solution (Sigma) pH = 4.2 for 10 min. After staining, the cells were washed with PBS for 5 min in order to remove unbound dye.

#### 2.2.3. Adipogenic Differentiation

For adipogenic differentiation, rabbit SMSC were plated in a density of 30,000 cells/cm^2^ in standard DMEM (low glucose) media and cultured until the cells reached 100% confluency. Subsequently, the media were replaced by adipogenic media adapted from Lee et al. [11], which contained DMEM (25 mM glucose), 10% FBS, 1% penicillin/streptomycin, 500 *μ*M IBMX (3-isobutyl-1-methylxanthine, Gibco, Herlev, Denmark), 1 *μ*M dexamethasone, 200 *μ*M indomethacin, and 10 *μ*g/ml insulin (Sigma, Brøndby, Denmark). Adipogenic media were changed every 2-3 days for 21 days, and cells were cultured in 37°C and 5% CO_2_.

*(1) Oil Red O Staining*. After 21 days of adipogenic differentiation, rabbit SMSC were stained with Oil Red O in order to assess adipocyte formation. Briefly, the cells were washed with PBS and fixed with 4% paraformaldehyde at room temperature for 10 min. Subsequently, the cells were washed with 3% isopropanol and incubated with 25 mg Oil Red O (Sigma) in 5 ml of 60% isopropanol and 3.35 ml H_2_O for 1 h.

### 2.3. RNA Isolation and Quantitative PCR

Total RNA was isolated from the cells using TRIzol® reagent (Invitrogen, Tastrup, Denmark) according to manufacturer's protocol. After 21 days of differentiation, the samples were dissociated in TRIzol using the gentleMACS Dissociator (Miltenyi Biotec, Lund, Sweden). cDNA was synthetized from 1 *μ*g of total RNA using a RevertAid H Minus First Strand cDNA Synthesis Kit (Thermo Scientific, MA, USA). Primers were designed using Primer-BLAST software. Primer sequences are described in Table 1 (Supplementary Figures available here). Real-time PCR was performed in the StepOnePlus Real-Time PCR System (Applied Biosystems) using Fast SYBR® Green Mix (Applied Biosystems, Foster City, CA, USA). Each sample was run in triplicates. The results were calculated using ΔCt method. The data are presented as 2−ΔΔCt, giving the relative expression change between day 0 and day 21.

### 2.4. Certification

The regional board for animal protection approved the experiments with the decision from 9 March 2013 with additional modifications at 21 August and 11 September 2015 (G-13/75).

### 2.5. Anesthesia

The rabbits received ketamine (35 mg/kg) combined with medetomidine (0.25 mg/kg) by intramuscular injection. During the surgery, Ringer's solution (10 ml/kg/h) was given through an intravenous access in the marginal ear vein. Anesthesia was supplemented with 0.5–2% isoflurane (double facemask with spontaneous ventilation, FiO_2_ > 0.4). Heart rate and blood oxygen saturation were monitored. Prior to surgery and 3 days after, the rabbits received carprofen, a nonsteroidal anti-inflammatory drug (4 mg/kg s.c.) as an analgesic and Baytril (enrofloxacin) 2.5% (0.4 ml/kg) as an antibiotic.

### 2.6. Operation

**Figure 1:**
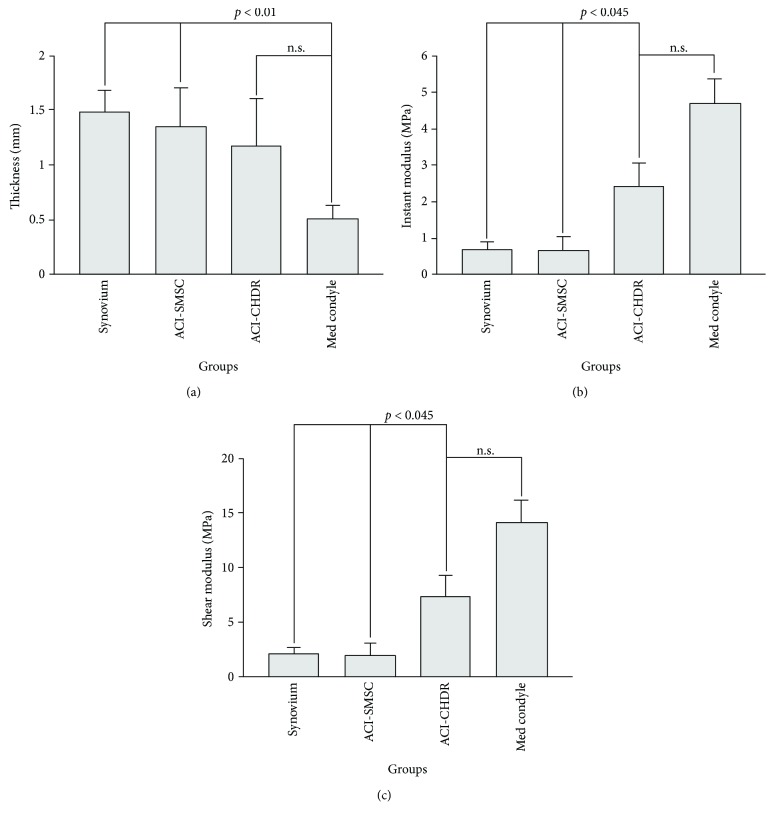
(a) Cartilage thickness. The cartilage thickness differs not significantly between the groups but is higher than normal caused by the technique of defect preparation (n.s.: not significant). (b) Instantaneous or instant modulus. The instant modulus is significantly higher in the group treated with amplified chondrocytes compared to the other intervention groups (n.s.: not significant). (c) Shear modulus. The shear modulus reached significantly higher values in the group treated with amplified chondrocytes compared to the other intervention groups (n.s.: not significant).

Female New Zealand white rabbits were obtained from Charles River (Sulzfeld, Germany). The animals were kept applying specific pathogen-free conditions and controlled room temperature. Acclimatization lasted 14 days. The animals were housed in cages with unrestricted water and food supply and a typical day/night rhythm. By the time of operation, the animals had reached a bodyweight of approximately 3.5 to 4.2 kg with closed growth plates. After shaving and disinfection, both knee joints were opened by a central skin cut and a medial parapatellar arthrotomy. Following this, the patella was laterally displaced. Hereafter, the Hoffa fat pad with the synovium was partially resected, and full-thickness cartilage lesions were prepared in the central medial femoral condyle using a drill with a 3.5 mm diameter and a stop at 2 mm depth. Attention was paid on an exact vertical angle for the drilling direction. Considering an average cartilage height of 0.5 mm (Figure 1(a)), the subchondral bone plate was opened. The defects of the control group were covered with a synovium flap, matching the prepared lesion size, and fixed with compression and fibrin glue (Baxter, Unterschleißheim, Germany). For the groups treated with cells, matching flaps of a bilayered collagen type I/III scaffold (Chondro-Gide, Geistlich, Pharma AG, Wolhusen, Switzerland) were prepared, passively seeded with cells (porous side, 400,000 cells per defect; 15 min adhesion time) and fixed as described for the synovium flaps. For inoculation, a cell suspension of 40,000,000 cells/ml was prepared and 10 *μ*l dropped on the tailored scaffold. After transplant fixation, the patella was relocated and the joint moved followed by a visual control of the correct transplant location. Hereafter, the arthrotomy and the skin were sutured separately. Wounds were sealed using an aluminium spray.

We compared 4 groups (synovium flap, nontreated uninjured cartilage, transplantation of amplified chondrocytes, or SMSC) and included 6 operated knees in each group (12 rabbits). The knees were randomly assigned to each group by using a random number generator. The animals were sacrificed after 6 weeks and the knees explanted and first biomechanically tested. After this, specimens were histologically analyzed. In a pilot study comparing the implantation of a collagen scaffold with and without chondrocytes, significant differences were found after 3, 6, 12, and 24 weeks (Oliver Huwert, unpublished data). The differences, however, declined from 7 to 5.4 points on a summary scale, evaluating macroscopic and histological scoring (Supplementary Figure 1). This was probably caused by the natural repair capacity of cartilage defects in rabbits and forced the focus on the 6-week time point.

### 2.7. Clinical Evaluation of Rabbits

The rabbits mobilized themselves immediately after weaning with full-weight bearing. They had no clinical signs of pain. They were closely observed one week until wound healing and had no signs of infection. The rabbits did not limp longer than 2-3 days having soon normal, species-specific moving patterns.

### 2.8. Biomechanical Evaluation

To define mechanical parameters, the multiaxial testing unit Mach-1 Model V500css (Biomomentum, Montreal, Canada) for automated normal alignment and indentation mapping with a multiple-axis load cell by Honeywell Mod. 34 (Honeywell, New Jersey, USA), a Newport Motion Controller ESP 301 (Newport, Irvine, USA), and an indenter with spherical geometry of 1 mm diameter was employed. For the biomechanical investigations, 10 different positions on the sample were examined. The positions were selected at the mapping module of Mach-1 Software (Biomomentum, Montreal). Five positions were selected on the healthy part of the sample (left condyle in Supplementary Figure 4A), and five positions were selected on the defective part of the sample (right condyle in Supplementary Figure 4A). To avoid dehydration of the cartilage during the measurements, the sample was moisturized several times with phosphate-buffered saline (PBS).

#### 2.8.1. Automated Indentation Mapping

For the automated indentation mapping, the following parameters were set in Mach-1 Motion: *z*-contact velocity of 0.5 mm/s, a contact criteria of 0.1015 N, and a scanning grid of 0.5 mm. The indentation amplitude was set to 0.2 mm and the indentation velocity to 0.2 mm/s. The relaxation time was set to 5 s.

#### 2.8.2. Automated Thickness Mapping

Thickness was mapped with the needle technique [12] by replacing the spherical indenter with a 27G × 3/4″ intradermal needle (B. Braun, Melsungen, Germany). The following parameters were input into the Mach-1 Motion Software: stage velocity of 0.5 mm/s; contact criteria of 4 N, and stage repositioning of 2x load resolution. The needle on the mechanical tester was directed vertically towards the sample at a constant speed until the cartilage surface was penetrated and the needle stopped at the subchondral bone edge.

#### 2.8.3. Data Processing

The findings were analyzed using the software Mach-1 Analysis Version 4.1.0.17 (Biomomentum, Montreal) and Origin 9.1 Professional (Origin Lab, Northampton, USA). The evaluation methods used were according to Sim et al. [13]. Using automated thickness mapping results, the cartilage thickness was calculated at each position from the difference between the vertical position of the surface (where the load starts to increase) and the position of the cartilage/bone interface (corresponding to the first inflection point in the displacement/force curve) (see Supplementary Figure 4B). The instantaneous modulus (IM or instant or Young's modulus) at each position was obtained by fitting the load-displacement curve (with corresponding thickness and effective Poisson ratio of 0.5) to an elastic model for indentation [14] (see following equation).

(1) $$\begin{array}{c} IM=\frac{P}{H}\cdot \frac{1-v^{2}}{2ak\cdot \left(\left(a/h\right)v\right)}, \end{array}$$

with *P* = load, *H* = indentation depth, *a* = radius of the contact region, *ν* = Poisson's ratio, and *k* = correction factor dependent on *a*/*h* and *ν.*

The shear modulus (G) is calculated as follows:

(2) $$\begin{array}{c} G=\frac{E}{2\left(1+v\right)}. \end{array}$$

An example for a typical strain-relaxation curve is provided in the Supplementary Material (Figure 4C).

### 2.9. Histology

After biomechanical testing, specimens were fixed in 4% buffered formaldehyde solution. A thin-section technique facilitated the preparation of the slides. The longitudinally sectioned specimens were decalcified with ethylene-diamine-tetra-acetic acid, dehydrated with ethanol, degreased with xylene substitute (Histoclear®), and then embedded in paraffin. The sections were made using a rotary microtome (HM 340E, Microm, Thermo Fisher Scientific Inc., Waltham, MA, USA). They were stained with hematoxylin-eosin and Safranin-O. The image analysis of the sections was done utilizing a light microscope (KS300, Carl Zeiss Ltd., Oberkochen, Germany) associated with a picture analysis unit (Axioplan, Carl Zeiss Vision Ltd., Oberkochen, Germany). Representative slides were selected and blindly evaluated by two different examiners, determining the OARSI score [15]. The average and the median were calculated and used for statistical group comparisons.

### 2.10. Immunohistology

For collagen type I, type II, and type X immunohistochemistry, sections were incubated for 30 min with 5% normal goat serum, followed by incubation with a 1 : 50 monoclonal mouse anticollagen type I antibody (MAB3391, Clone 5D8-G9, Chemicon, Hofheim, Germany), a 1 : 50 monoclonal mouse anticollagen type II antibody (MAB8887, Clone 6B3, Chemicon), or a 1 : 500 monoclonal mouse anticollagen type X antibody (Clone COL-10, C7974, Sigma, Taufkirchen, Germany) for 2 h, three washings with PBS, and incubation with a biotin-labelled goat antimouse immunoglobulin for 30 min (Acris, Herford, Germany). Afterwards, sections were incubated with avidin for 30 min and with 3-amino-9-ethylcarbazole (AEC) substrate for 10 min. Representative slides were selected, blinded, and evaluated by two different examiners, determining the Remmele-Stegner Score [16] and comparing positive and negative stains. The average and the median were calculated and used for statistical group comparisons.

### 2.11. Statistics

All values were expressed as mean ± standard error of the mean. Regarding the scores and all numerical values (if *n* < 5), statistical significance was tested nonparametrically primarily using the Mann–Whitney *U* test. Multiple comparisons were calculated using a post hoc statistics with the Kruskall-Wallis *H* test. Probability distributions of samples with *n* ≥ 5 were analyzed by a Kolmogorov–Smirnov test. Normally distributed samples were then compared using a *t*-test, otherwise the samples were nonparametrically compared as indicated. Statistical significance was defined as *p* < 0.05.

## 3. Results

### 3.1. Cell Characterization

**Figure 2:**
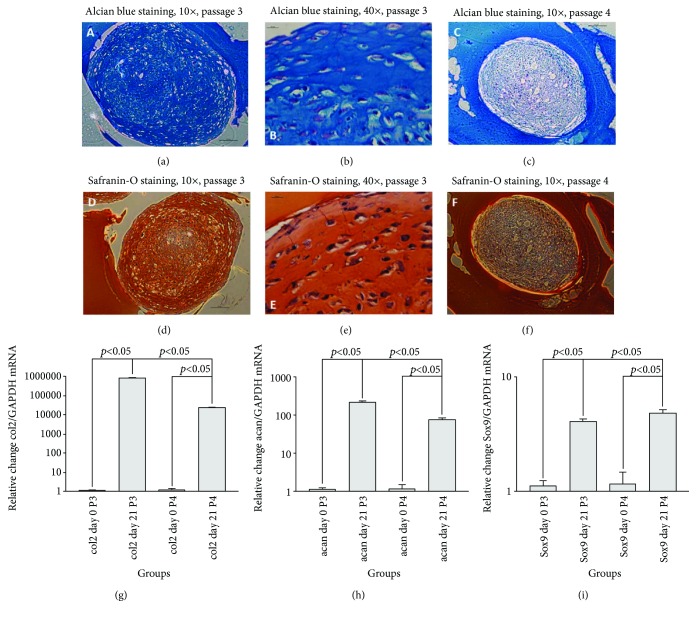
Rabbit SMSC have the ability to undergo chondrogenic differentiation in vitro in a pellet culture. Staining with Alcian blue (upper row) and Safranin-O (lower row) indicates a successful production of glycosaminoglycans after 21 days. Chondrogenic differentiation capacity declined remarkably with a further passage. Specimens were embedded in gelatin before staining. Quantitative PCR revealed mRNA formation of typical cartilage markers such as collagen type II (g), Aggrecan (h), and Sox9 (i) in both passage 3 and 4 (*n* = 3, *p* < 0.05).

Rabbit synovial mesenchymal stem cells (SMSC) differentiated readily into chondrocytes in pellet culture after 3 weeks. Alcian blue staining of glycosaminoglycans of the pellets is shown in Figures 2(a) and 2(b). Similar staining pattern of cartilage by Safranin-O was observed (Figures 2(d) and 2(e)). The cells exhibited a partially fibroblast-like phenotype. Chondrogenic differentiation capacity declined remarkably after one further passage of the cells (Figures 2(c) and 2(f)).

Furthermore, quantitative RT-PCR revealed overexpression of cartilage mRNA markers during chondrocyte differentiation of SMSC. As seen in Figures 2(g)–2(i), collagen type II (col2), Aggrecan (acan), and Sox9 mRNA were significantly induced, and this was observed in both p3 and p4 cells. However, the response was lower in p4 compared to p3 cells.

Osteogenic differentiation of SMSC was evidenced by typical morphological changes (Supplementary Figures 2A and 2B). Alizarin Red staining visualized the formation of mineralized extracellular matrix. However, both the intensity of Alizarin Red stain and the lack of alkaline phosphatase expression indicated a limited capacity for osteoblastic differentiation (Supplementary Figures 2C and 2E). Quantitative RT-PCR analysis corroborated this finding, showing marginally increased collagen type I (col1) and osteocalcin (OCN) mRNA expression after 21 days (Supplementary Figure 2D). Similar to adipocyte differentiation, passage 4 cells had very limited osteoblastic differentiation capacity.

Rabbit SMSC were also able to differentiate in adipocytes, demonstrated by an Oil Red O stain (Supplementary Figures 3A and 3B). Adipocyte's differentiation was associated with accumulation of small lipid droplets and the induction of adipogenic genes such as PPARG (peroxisome proliferator-activated receptor gamma) and AN (adiponectin) (Supplementary Figure 3C). Together with a decreasing proliferation rate, p4 cells almost lost their adipogenic differentiation capacity (data not shown).

### 3.2. Gross Evaluation

The evaluation of the macroscopic degree of recovery using the ICRS subscore for defect filling [17] showed successful healing of the medial condyle lesions reaching values ranging below 3. We did not observe differences between the intervention groups (Table 1). All knees of the control group without intervention had naturally no cartilage damage (ICRS subscore = 0). The results are not separately reported, because there was no area of healing or lesion. Furthermore, the percentage of surface in the lesion containing repair tissue corresponding with the adjacent natural cartilage was evaluated as “area of healing.” Most of the surface was covered by repair tissue, but we did not detect differences between the groups evaluated 6 weeks postinjury (Table 1).

The evaluation of the macroscopic degree of healing using the ICRS score and the area of healing did not show differences between the groups after 6 weeks. Each group was statistically compared to every other group; the results are indicated by n.s. (not significant).

### 3.3. Biomechanical Evaluation

#### 3.3.1. Thickness

The thickness of the uninjured reference cartilage of the medial femoral condyle was 0.50 ± 0.12 mm. There were no statistically significant differences between the cartilage thickness obtained in the intervention groups: 1.47 ± 0.20 mm for the synovium group, 1.35 ± 0.36 mm for transplanted SMSC, and 1.17 ± 0.44 mm for transplanted amplified chondrocytes (Figure 1(a)). The comparison between the uninjured control and each of the synovium group or the SMSC group revealed significant statistical differences (*p* < 0.01); however, the direct statistical comparison of the chondrocyte and the control group failed to reach significance (*p* = 0.06).

#### 3.3.2. Instantaneous or Instant Modulus (IM)

The instantaneous or instant modulus describes mechanical stress resistance of cartilage and correlates with osteoarthritic changes [13]. Normal cartilage is predicted to have high IM values, correlating with the instant modulus that was highest in the uninjured control group reaching 4.69 ± 0.68 MPa. There were no statistically significant differences between the groups with a transplanted piece of synovium (1.48 ± 0.20 MPa) and transplanted SMSC (1.35 ± 0.358 MPa, *p* = 0.85). However, both groups had a lower instant modulus than the control group (*p* < 0.001) and the group with transplanted amplified chondrocytes (2.42 ± 0.66 MPa, *p* < 0.05). Although numerically lower, the instant modulus of the chondrocyte group was not significantly different from the control group without defect (*p* = 0.06, Figure 2(b)).

### 3.4. Shear Modulus

The collagen content influences the shear modulus, which describes the material's response to shear stress and provides a functional measure of cartilage health [18]. The pattern of this analysis resembles the instant modulus. The shear modulus was highest in the uninjured control group reaching 14.09 ± 2.05 MPa. There was no statistically significant difference between the group with a transplanted piece of synovium (2.06 ± 0.63 MPa) and transplanted SMSC (1.90 ± 1.16, *p* = 0.85). However, both groups had a lower instant modulus than the control group (*p* < 0.001) and the group with transplanted amplified chondrocytes (7.27 ± 1.97 MPa, *p* < 0.05). Although numerically lower, the instant modulus of the chondrocyte group did not statistically significantly differ from the control group without a lesion (*p* = 0.06, Figure 2(c)).

### 3.5. Histological Evaluation

**Figure 3:**
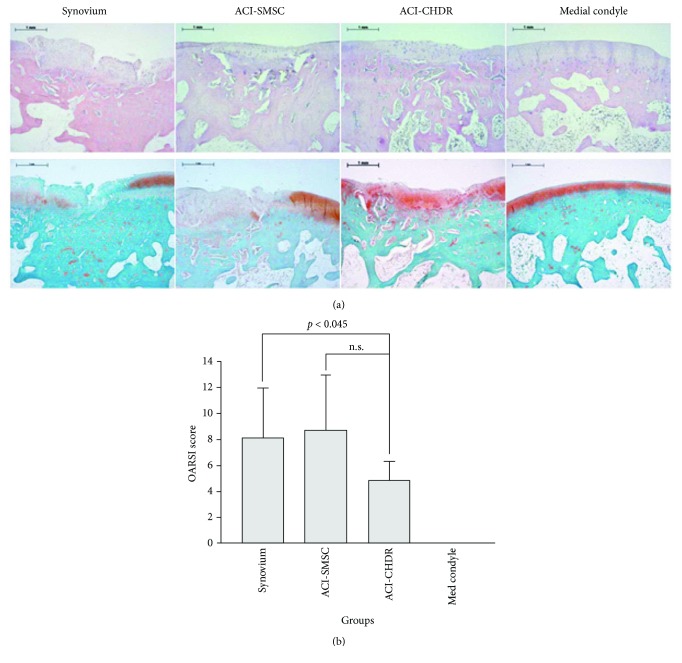
(a) HE (upper row) and Safranin-O (lower row) staining of the medial condyle showing the defect region with regenerative tissue following the different interventions. The red color indicates the presence of glycosaminoglycans. (b) The OARSI score is lower in the lesions treated with amplified chondrocytes compared to negative controls (n.s.: not significant).

For qualitative histological evaluation of the regenerated tissue, the specimens were stained by HE and Safranin-O (Figure 3(a)). The stains confirmed uniform defect preparation and defect filling as well as the expression of glycosaminoglycans. The bonding at defect edges had partially a very high quality independent of the intervention group. The defects were slightly conically shaped, corresponding with the drill, which was used for defect preparation. There were no signs of immunological reactions against the implanted material evidenced by the absence of macrophages or giant cells.

The OARSI score was employed to obtain quantitative comparison. All uninjured cartilage areas had no sign of osteoarthritis (OARSI score = 0), which was significantly better than the scores of all intervention groups. The lowest scores were observed following treatment with amplified chondrocytes reaching 4.88 ± 1.43 points, compared to 8.11 ± 3.84 points (*p* < 0.05) in the synovium group and 8.71 ± 4.19 points (*p* = 0.05) in defects treated with amplified SMSC. Only the comparison with the synovium group reached statistical significance level (Figure 3(b)).

### 3.6. Immunohistology

The quantitative evaluation of the collagen type I, II, and X expression using the Remmele-Stegner Score did not show differences between the intervention groups after 6 weeks (Table 2), but all collagen types were expressed in the different types of original or regenerative cartilage tissue.

The evaluation of the collagen type I, II, and X expression quantified using the Remmele-Stegner Score did not show differences between the groups after 6 weeks. Each group was statistically compared for each examined collagen to every other group (n.s. (not significant)).

**Figure 4:**
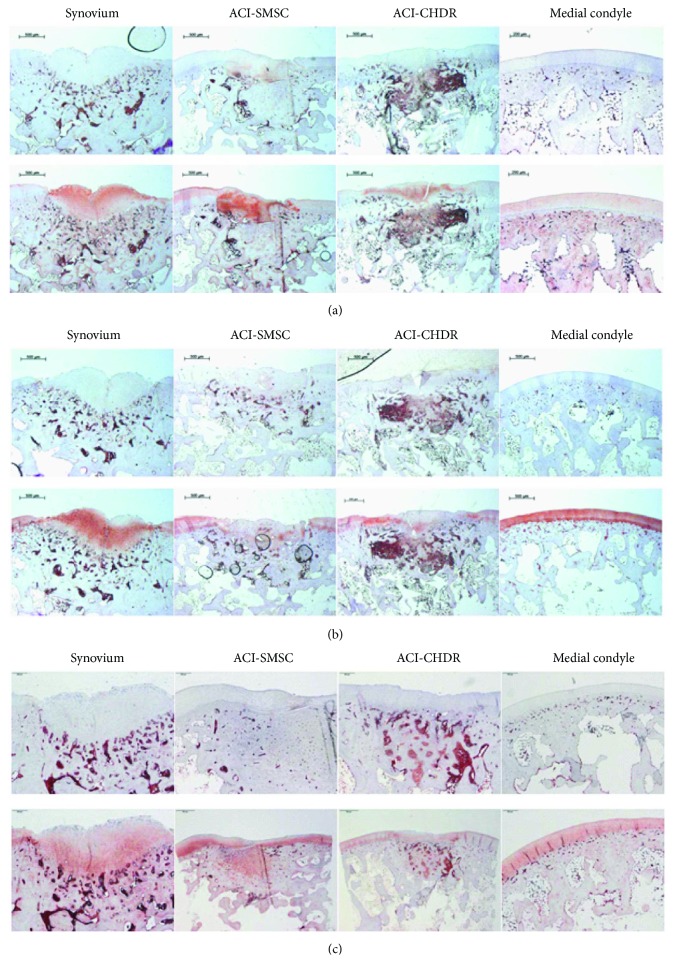
(a) Collagen type I staining of the medial condyle showing the defect region with regenerative tissue following the different interventions. The slides of the upper row were stained with an isotype control antibody; the slides of the lower row were stained using the specific antibody. Red color indicates positive staining. (b) Collagen type II staining of the medial condyle showing the defect region with regenerative tissue following the different interventions. The slides of the upper row were stained with an isotype control antibody; the slides of the lower row were stained using the specific antibody. Red color indicates positive staining. (c) Collagen type X staining of the medial condyle showing the defect region with regenerative tissue following the different interventions. The slides of the upper row were stained with an isotype control antibody; the slides of the lower row were stained using the specific antibody. Red color indicates positive staining.

The levels of staining quality and intensity were comparable for collagen types I and II and lower for collagen type X in the intervention groups.  Figure 4 shows representative slides for collagen types I (a), II (b), and X (c), comparing the staining of the specific antibodies with their isotype controls.

## 4. Discussion

The main finding of this study is that rabbit synovium-derived stromal (mesenchymal) stem cells (SMSC) can differentiate into the adipogenic, osteogenic, and chondrogenic lineage in vitro; however, SMSC are most suitable to form cartilage. Undifferentiated SMSC are eligible for matrix-associated implantation in a defect model in rabbits; however, the gained biomechanical stability of the regenerated cartilage and its quality, evaluated by a validated histological osteoarthritis score, was lesser compared to the current standard protocol utilizing amplified chondrocytes.

Numerous studies have highlighted the chondrogenic phenotype of SMSC [5, 6], which indicate an extraordinary suitability for natural [19] and surgically induced cartilage repair purposes [10, 20]. This was supported by findings in pathological processes, showing that synovial chondrogenesis leads to synovial chondromatosis [21]. A direct comparison of mesenchymal stem cells of different origin pointed out significant differences and a superiority of SMSC for cartilage formation [22]. This data is in line with our findings, demonstrating formation of high-quality cartilage with expression of glycosaminoglycans and collagen type II using rabbit SMSC. In contrast, the ability for adipogenic and osteogenic differentiation of these cells was limited. Lee et al. described similar findings and subjoined that the expression of certain surface markers, such as CD29 and CD90, differs between mesenchymal stem cells of human and rabbit origin and that they vary regarding their phenotype [11]. Despite this, different in vivo models demonstrated comparable functioning and success for rabbit SMSC applications [10, 11]. Beyond that, these cells supported successful cartilage regeneration in animal models of other species [20, 23] and in clinical applications in humans. SMSC were for instance successfully used for arthroscopically assisted cartilage repair resulting in improved MRI features, histology, and clinical outcome [24]. However, the study included only 10 patients without standardized follow-up and control group. By demonstrating that a single injection of SMSC was ineffective, but weekly injections in rat knees had significant chondroprotective effects, Ozeki et al. pointed out that the modus of application possesses a decisive influence on treatment attainment [25]. Pei et al. demonstrated the successful usage of SMSC seeded into nonwoven polyglycolic acid mesh in combination with a synthetic bone substitute to repair osteochondral defects in rabbit knee joints [10]. However, the cells were first treated in an incubator 1 month before implantation using a growth factor cocktail. Thereafter, the implants were biochemically, biomechanically, and histologically characterized, showing that the maturation prior to implantation improved the construct. The basis for the design of the presented study was the need for easy-to-use protocols for clinical applications. Therefore, the procedure was the same as a standard protocol for the autologous chondrocyte implantation, in which the chondrocytes were substituted with SMSC. Otherwise, the cells had the same starting conditions regarding passage, origin from the same rabbit, allogenic transplantation, and viability. The chondrocytes, currently the “gold standard,” served at the same time as a control. Based on the very promising in vitro data of our own experiments and the results published by other groups regarding their chondrogenic predifferentiation, we expected to see similar results, when the in vitro predifferentiation, which was to date applied by all other groups, was omitted [10]. This approach should avoid another characteristic problem of preformed constructs, the failure of sufficient boundary integration as observed by Fujie et al. [26]. A successful testing of this procedure would also be the prerequisite for a possible one-step protocol, which truly would improve the perspectives of this method. However, the undifferentiated SMSC failed to show the same efficiency in cartilage repair as chondrocytes. Although the height of the regenerated tissue in the defect was larger than that in all the intervention groups compared to natural cartilage, the biomechanical stress resistance was lower. Since the prepared defect was 2 mm and therefore larger than the natural cartilage height, this difference is obvious and similar between the intervention groups. The measured height of the regenerated tissue correlated with the defect depth, which indirectly confirmed the uniform configuration of the lesions. Neither gross evaluation nor regeneration height differed in the intervention groups, which directly leads to the conclusion that the actual relevant parameter for the quality evaluation of regenerative tissue is the instantaneous and the shear modulus, reflecting the biomechanical stress resistance. Similarly, this was described by an analysis using scaffold-free tissue-engineered constructs utilizing SMSC [27], which exhibited compressive properties similar to uninjured cartilage. However, also in this study, the constructs lacked the typical zonal configuration of mature cartilage, having inferior surface stiffness and water retention capacity. The lack of a directional differentiation environment may have caused this. Therefore, the biomechanics could possibly be improved significantly when combining SMSC in a scaffold with growth factors, inducing chondrogenic differentiation such as TGF*β*.

The collagen types I, II, and X characterize fibrocartilage and chondrocyte dedifferentiation, typical hyaline cartilage, and cartilage hypertrophy, respectively [28]. Although all collagen types were expressed without statistically significant differences between the intervention groups, differences were found comparing the level of collagen types. Whereas the quantity and quality for staining of collagen types I and II equaled each other, the level of collagen type X expression was least. This is a typical pattern for early cartilage repair [29], especially when the regeneration is associated with partial fibrocartilage formation.

Possible explanations for the less effectivity in cartilage defect healing are the original differentiation capacity of the synovial cells used. Caused by the anatomical conditions of a rabbit with very small dimensions, it is difficult to separate synovium from the rest of the surrounding tissue such as the Hoffa fat pad. By this, other types of mesenchymal stem cells derived from fat tissue or CD44+CD34− adventitial nonendothelial progenitor cells might have been mixed in the implanted stem cell population and have influenced the resulting cartilage-forming abilities [30]. The cells were also not labeled for later tracing purposes. Furthermore, the reported outcomes reflect only short-term results. The 6 weeks might simply have been not long enough to allow chondrogenic differentiation in vivo and adequate cartilage formation. However, an in vitro study using a transwell coculture has demonstrated that already after 7 days, SMSC could differentiate into chondrocytes forming typical chondron-like structures without applying biomechanical stimulation [6]. Moreover, biomechanical stimulation is expected to significantly support cartilage formation [31].

There are several possibilities to improve the results following SMSC transplantation. First, the cells could be predifferentiated during the amplification phase as shown before [10]. However, this complicates the preparation process prior to implantation and therefore challenges the production process. Furthermore, a different scaffold could be used, providing biomechanical characteristics or/and a growth factor-releasing mechanism, supporting spontaneous chondrogenic differentiation [32]. The study does not address the influence of time, which could lead to further maturation of the regenerative tissue under the influence of the natural environment. Furthermore, other groups have tested injection techniques of SMSC, showing successful treatment of osteoarthritis or cartilage lesions [20, 25, 33]. However, the functioning mechanism of this approach remains uncertain, because it is not clear whether the cells are enriched in the lesion and how the cells act.

## 5. Conclusion

Synovium-derived stem cells (SMSC) demonstrated a high chondrogenic potential in vitro. When used for matrix-associated implantation in a defect model in rabbits, undifferentiated SMSC showed macroscopic and immunohistological repair results comparable to the current standard utilizing amplified chondrocytes. However, biomechanical resistance and histological scoring were inferior. Considering the large evidence for the potential of SMSC in cartilage regeneration, the search for alternative protocols and conditions should continue. Furthermore, the study suggests that allogenic implantation is possible for both mesenchymal stem cells and chondrocytes, offering a significant simplification of the matrix-associated cell implantation.

## Figures and Tables

**Table 1:** Macroscopic evaluation.

Defect	Group	ICRS grading	*p* (versus others)	Area	*p* (versus others)
Synovium	1	2.2 ± 0.4	n.s.	96.8 ± 10.2	n.s.
ACI-SMSC	2	2.7 ± 0.8	n.s.	83.3 ± 30.3	n.s.
ACI-CHDR	3	2.7 ± 0.8	n.s.	79.2 ± 24.6	n.s.

**Table 2:** Quantitation of immunostaining using the Remmele-Stegner Score.

Defect	Collagen type I	*p*	Collagen type II	*p*	Collagen type X	*p*
Synovium	6.1 ± 1.7	n.s.	6.1 ± 3.2	n.s.	1.8 ± 2.9	n.s.
ACI-SMSC	5.5 ± 1.6	n.s.	4.8 ± 2.8	n.s.	2.2 ± 1.6	n.s.
ACI-CHDR	5.1 ± 0.9	n.s.	4.0 ± 1.3	n.s.	2.6 ± 2.1	n.s.

## Supplementary Materials

Supplementary Materials Supplementary Table 1: the used primer sequences. Supplementary Figures 4A–4C: a more detailed description of the biomechanical analysis. Supplementary Figure 2: osteogenic differentiation of rabbit SMSC. Supplementary Figure 3: a description of adipogenic differentiation of rabbit SMSC.
