# Supplementary material for: Comparison of Regenerative Tissue Quality following Matrix-Associated Cell Implantation Using Amplified Chondrocytes Compared to Synovium-Derived Stem Cells in a Rabbit Model for Cartilage Lesions
Source: Stem Cells Int. 2018 Apr 19;2018:4142031. doi: 10.1155/2018/4142031 (PMC5933044; doi:10.1155/2018/4142031)
Supplement: Supplementary Materials — Supplementary Table 1: the used primer sequences. Supplementary Figures 4A–4C: a more detailed description of the biomechanical analysis. Supplementary Figure 2: osteogenic differentiation of rabbit SMSC. Supplementary Figure 3: a description of adipogenic differentiation of rabbit SMSC. [file 4142031.f1.pptx]

## Slide 1
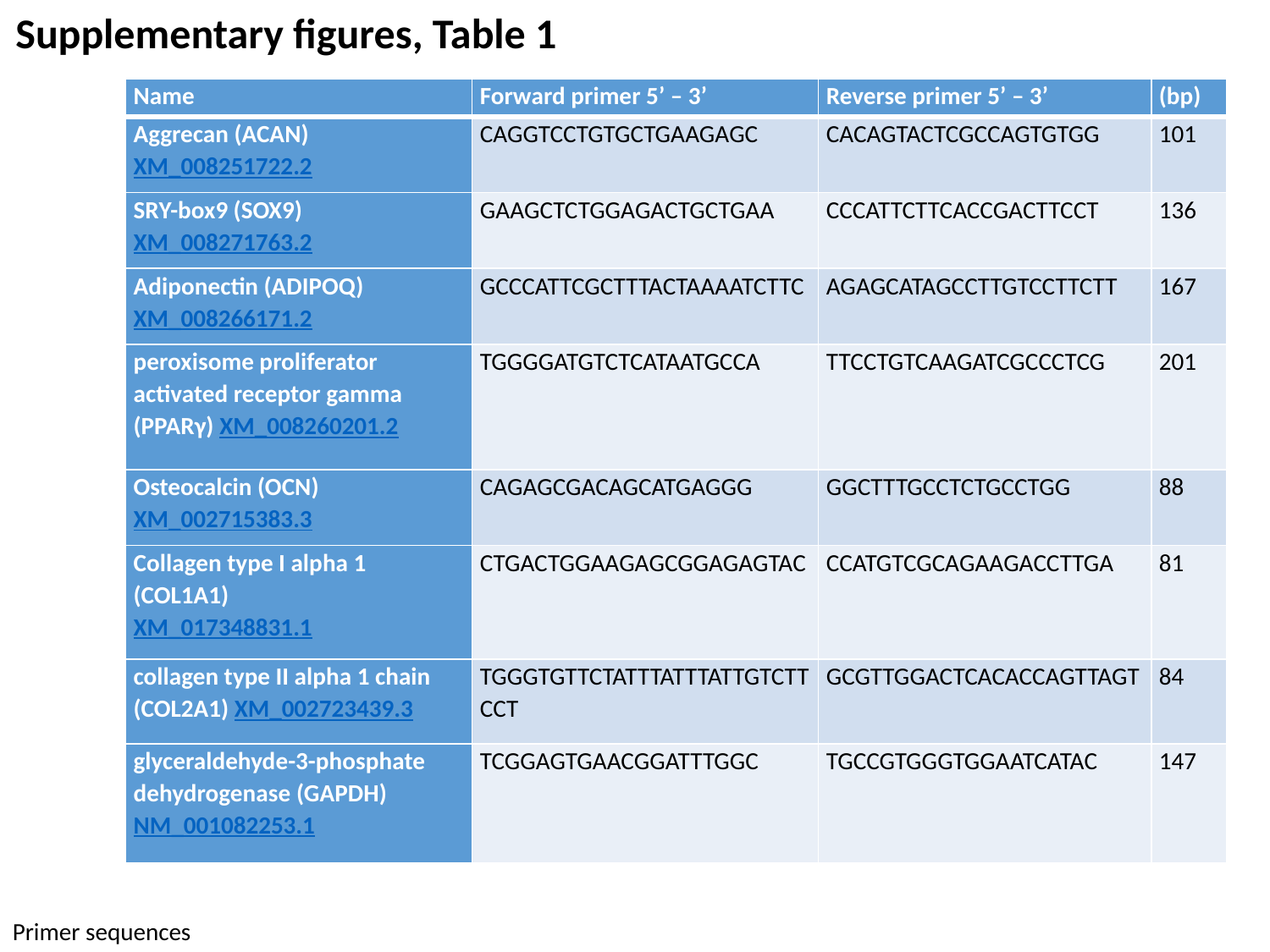

Supplementary figures, Table 1
| Name | Forward primer 5’ – 3’ | Reverse primer 5’ – 3’ | (bp) |
| --- | --- | --- | --- |
| Aggrecan (ACAN) XM\_008251722.2 | CAGGTCCTGTGCTGAAGAGC | CACAGTACTCGCCAGTGTGG | 101 |
| SRY-box9 (SOX9) XM\_008271763.2 | GAAGCTCTGGAGACTGCTGAA | CCCATTCTTCACCGACTTCCT | 136 |
| Adiponectin (ADIPOQ) XM\_008266171.2 | GCCCATTCGCTTTACTAAAATCTTC | AGAGCATAGCCTTGTCCTTCTT | 167 |
| peroxisome proliferator activated receptor gamma (PPARγ) XM\_008260201.2 | TGGGGATGTCTCATAATGCCA | TTCCTGTCAAGATCGCCCTCG | 201 |
| Osteocalcin (OCN) XM\_002715383.3 | CAGAGCGACAGCATGAGGG | GGCTTTGCCTCTGCCTGG | 88 |
| Collagen type I alpha 1 (COL1A1) XM\_017348831.1 | CTGACTGGAAGAGCGGAGAGTAC | CCATGTCGCAGAAGACCTTGA | 81 |
| collagen type II alpha 1 chain (COL2A1) XM\_002723439.3 | TGGGTGTTCTATTTATTTATTGTCTTCCT | GCGTTGGACTCACACCAGTTAGT | 84 |
| glyceraldehyde-3-phosphate dehydrogenase (GAPDH) NM\_001082253.1 | TCGGAGTGAACGGATTTGGC | TGCCGTGGGTGGAATCATAC | 147 |
Primer sequences

## Slide 2
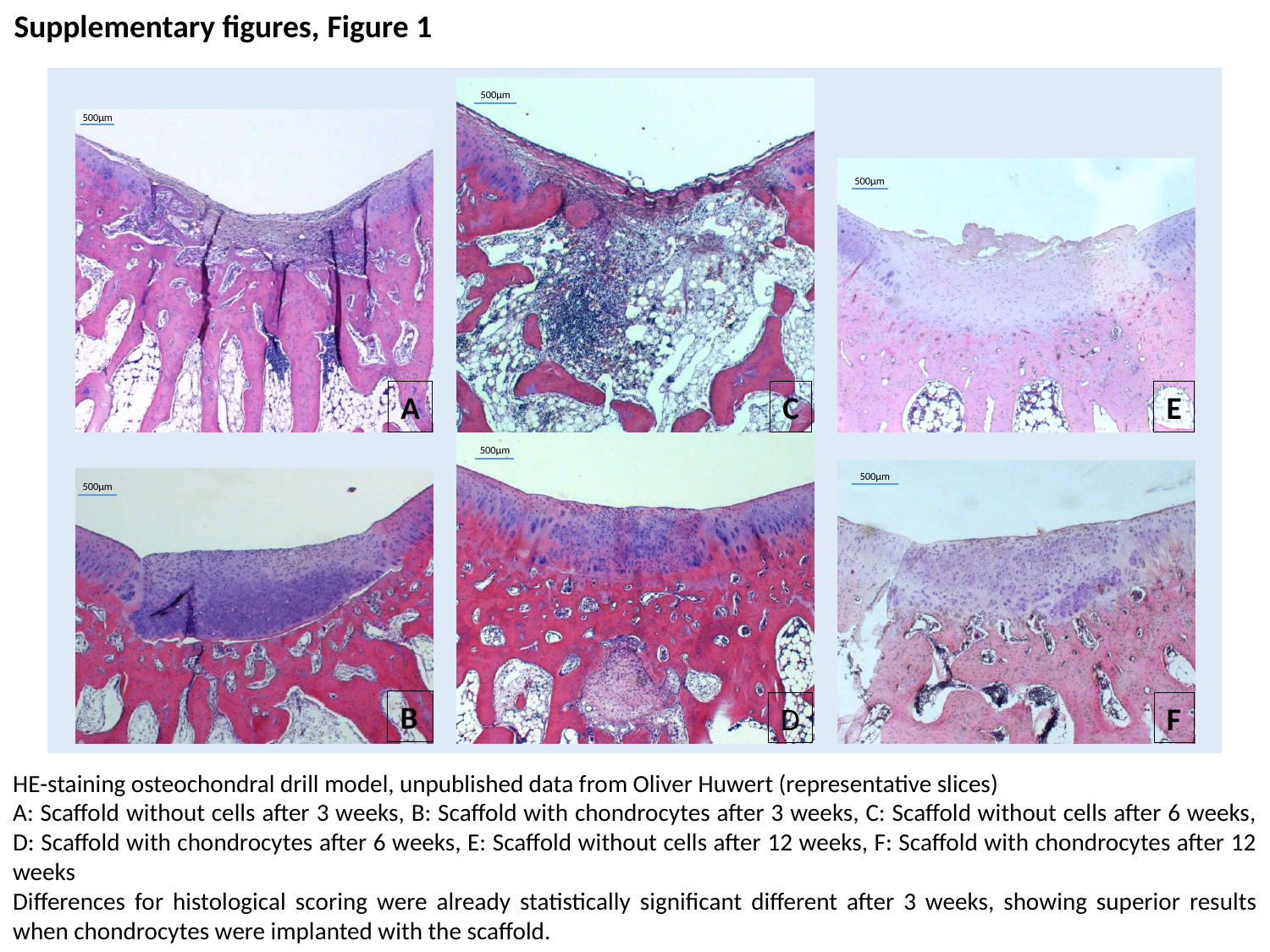

Supplementary figures, Figure 1
500µm
500µm
500µm
A
C
E
500µm
500µm
500µm
B
D
F
HE-staining osteochondral drill model, unpublished data from Oliver Huwert (representative slices)
A: Scaffold without cells after 3 weeks, B: Scaffold with chondrocytes after 3 weeks, C: Scaffold without cells after 6 weeks, D: Scaffold with chondrocytes after 6 weeks, E: Scaffold without cells after 12 weeks, F: Scaffold with chondrocytes after 12 weeks
Differences for histological scoring were already statistically significant different after 3 weeks, showing superior results when chondrocytes were implanted with the scaffold.

## Slide 3
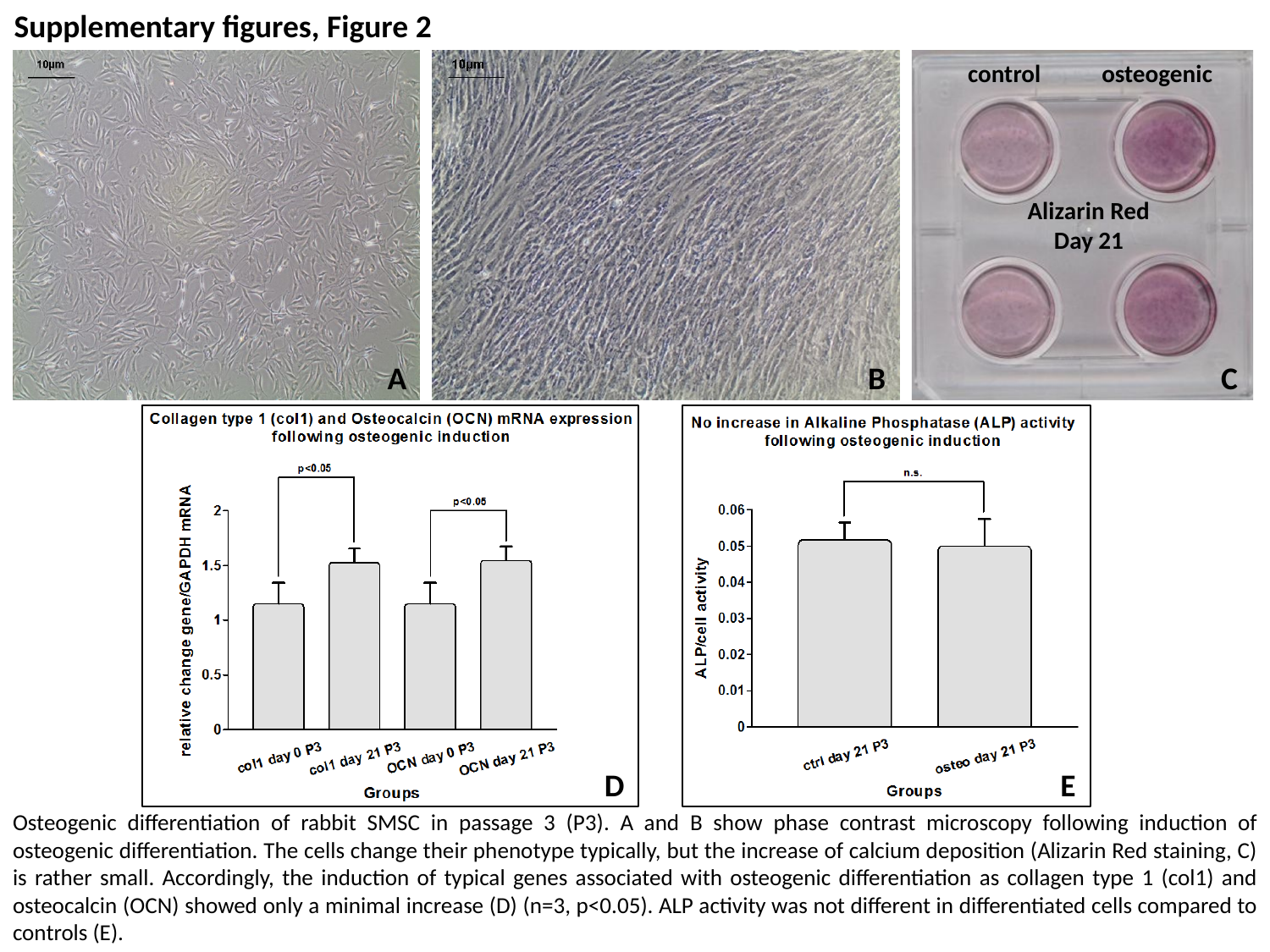

Supplementary figures, Figure 2
osteogenic
control
Alizarin Red
Day 21
A
B
C
D
E
Osteogenic differentiation of rabbit SMSC in passage 3 (P3). A and B show phase contrast microscopy following induction of osteogenic differentiation. The cells change their phenotype typically, but the increase of calcium deposition (Alizarin Red staining, C) is rather small. Accordingly, the induction of typical genes associated with osteogenic differentiation as collagen type 1 (col1) and osteocalcin (OCN) showed only a minimal increase (D) (n=3, p<0.05). ALP activity was not different in differentiated cells compared to controls (E).

## Slide 4
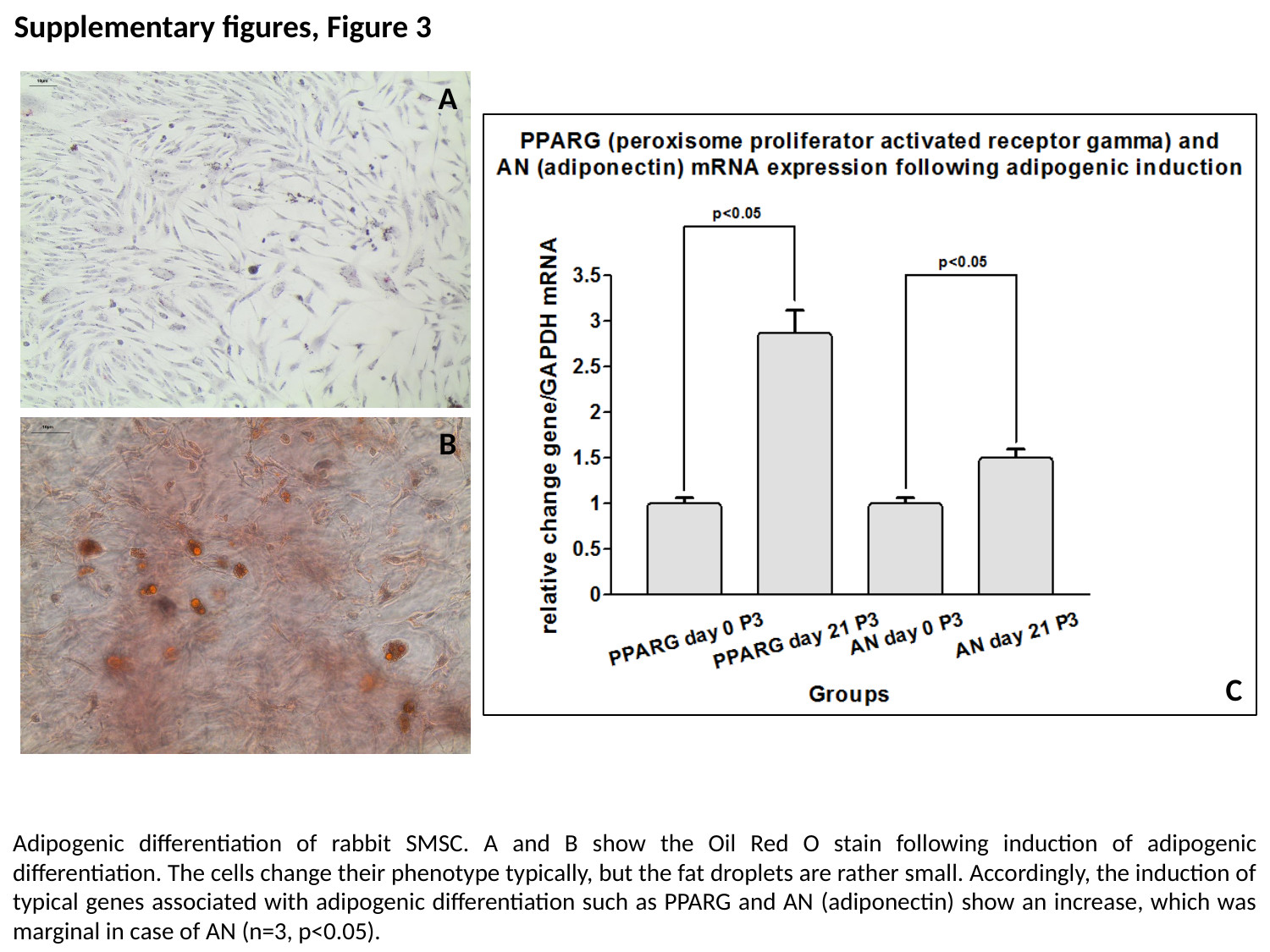

Supplementary figures, Figure 3
A
B
C
Adipogenic differentiation of rabbit SMSC. A and B show the Oil Red O stain following induction of adipogenic differentiation. The cells change their phenotype typically, but the fat droplets are rather small. Accordingly, the induction of typical genes associated with adipogenic differentiation such as PPARG and AN (adiponectin) show an increase, which was marginal in case of AN (n=3, p<0.05).

## Slide 5
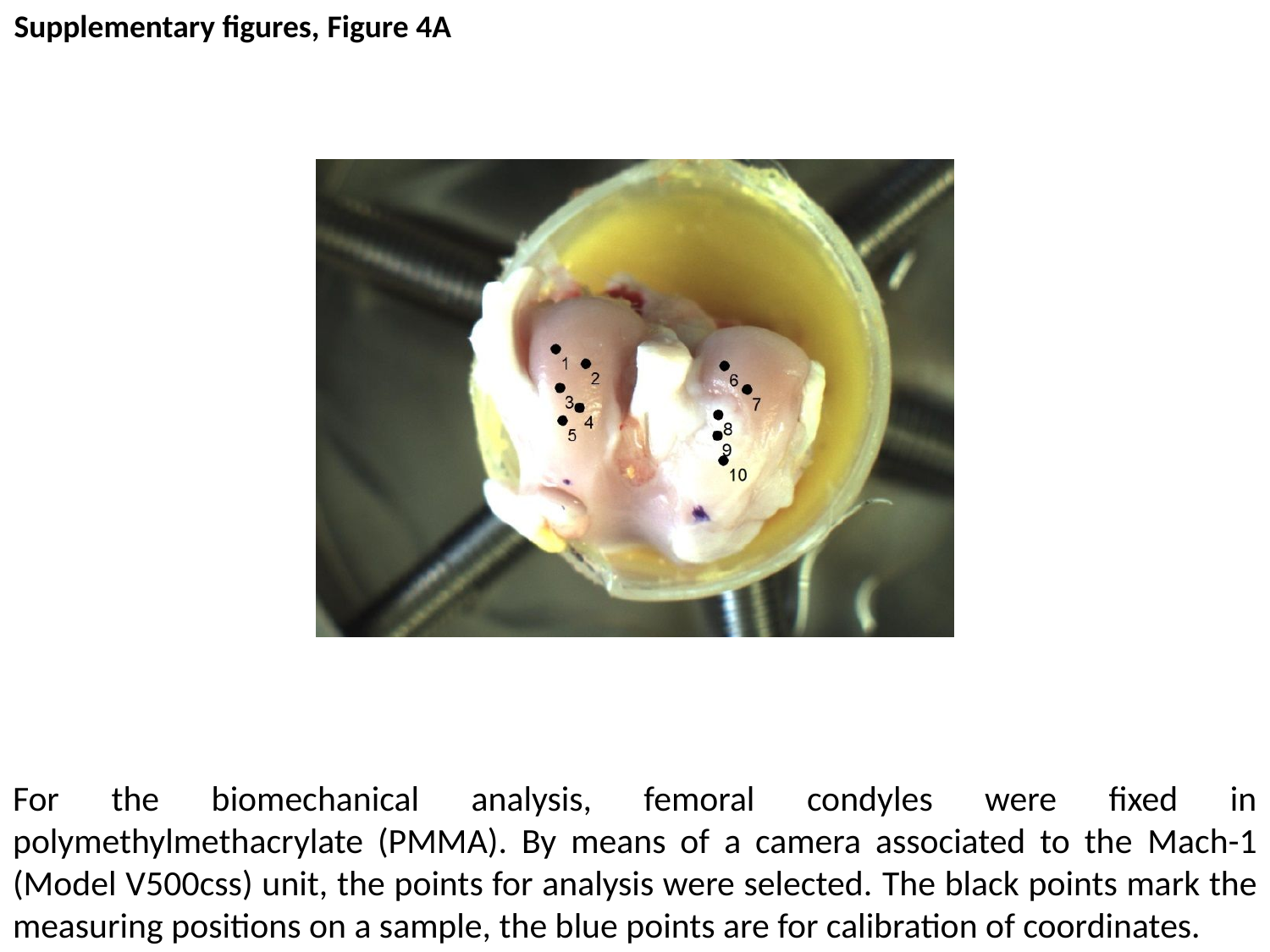

Supplementary figures, Figure 4A
For the biomechanical analysis, femoral condyles were fixed in polymethylmethacrylate (PMMA). By means of a camera associated to the Mach-1 (Model V500css) unit, the points for analysis were selected. The black points mark the measuring positions on a sample, the blue points are for calibration of coordinates.

## Slide 6
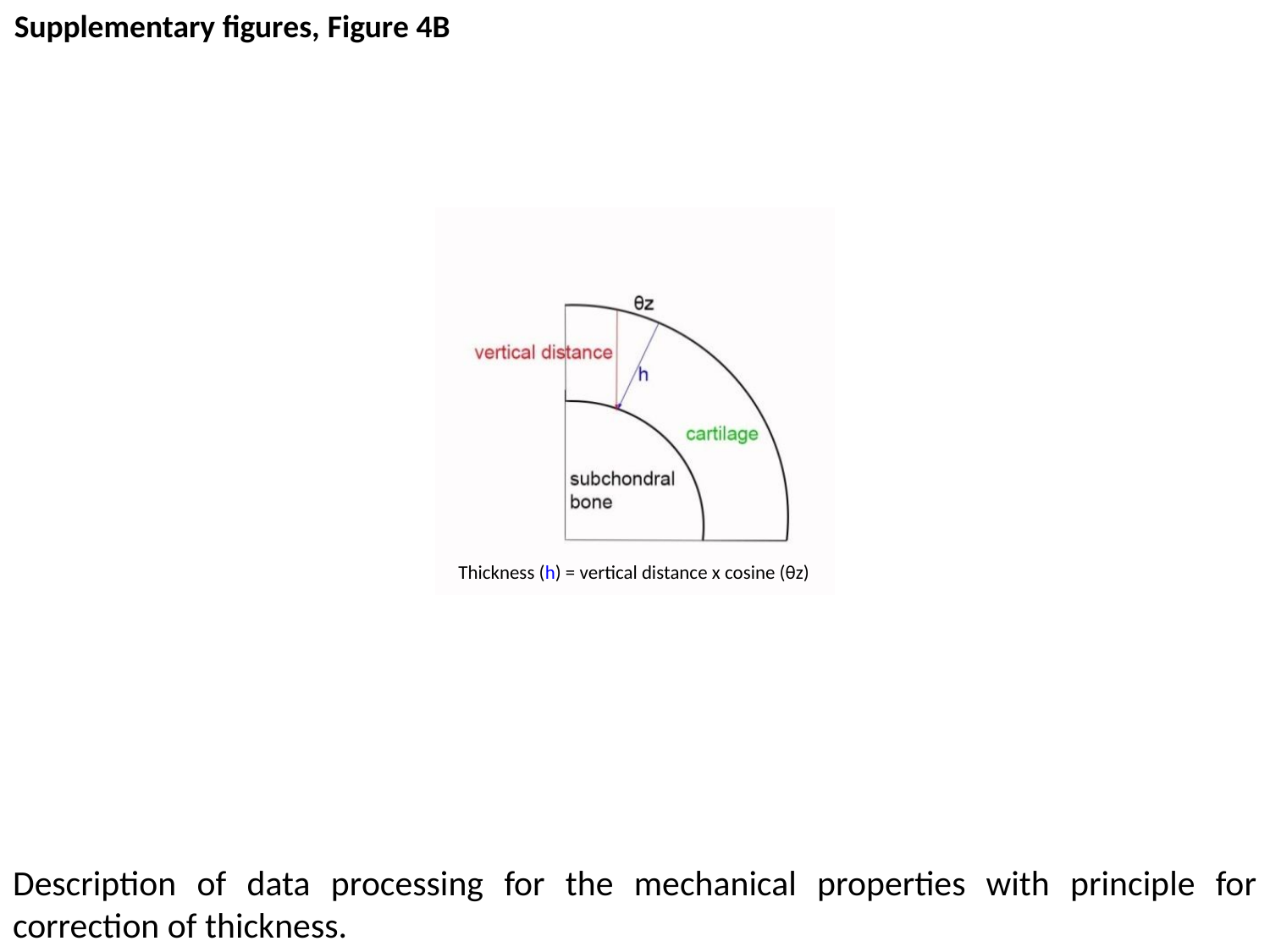

Supplementary figures, Figure 4B
Thickness (h) = vertical distance x cosine (θz)
Description of data processing for the mechanical properties with principle for correction of thickness.

## Slide 7
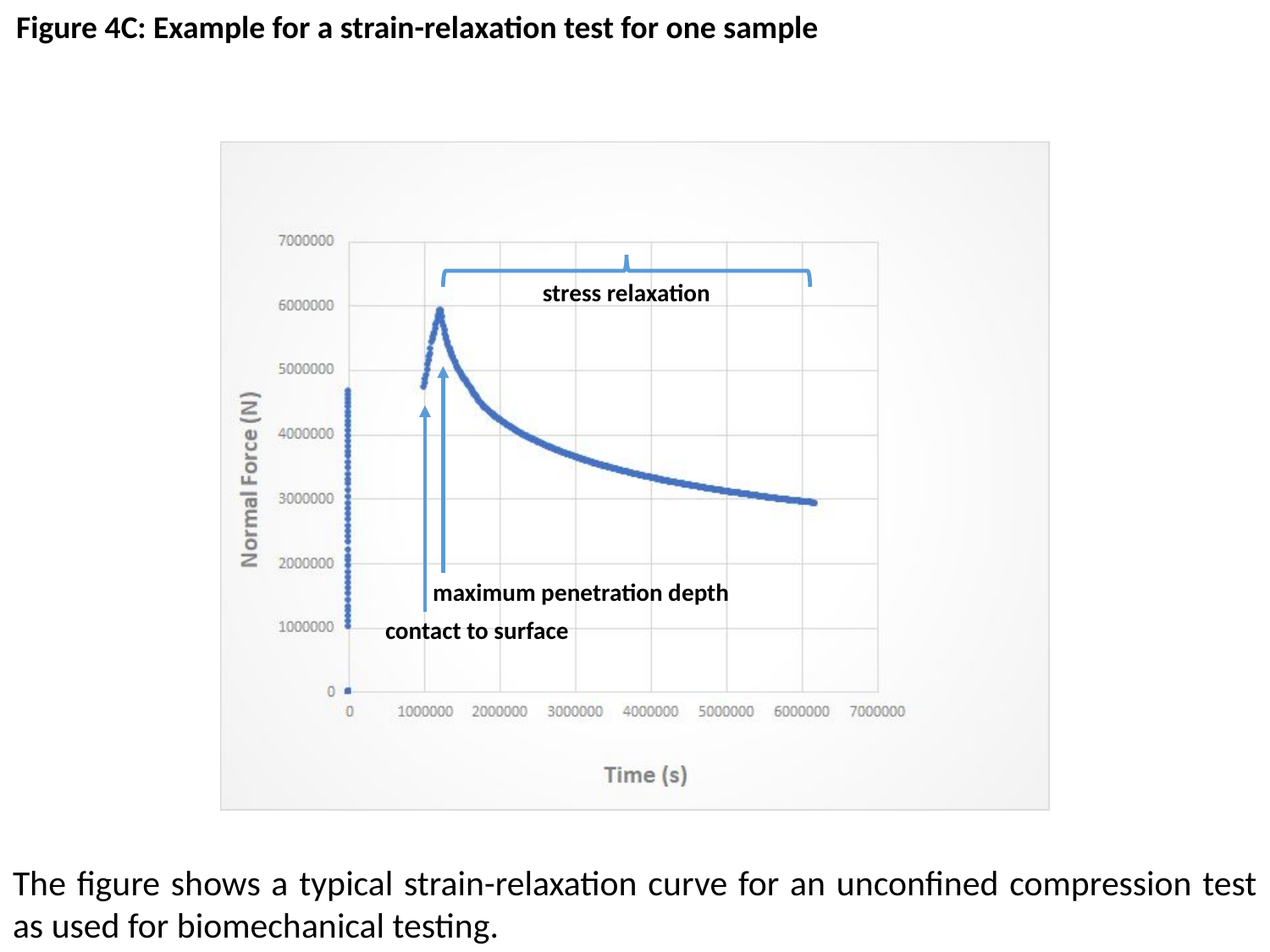

Figure 4C: Example for a strain-relaxation test for one sample
stress relaxation
maximum penetration depth
contact to surface
The figure shows a typical strain-relaxation curve for an unconfined compression test as used for biomechanical testing.
